# Enhanced fatty acid oxidation provides glioblastoma cells metabolic plasticity to accommodate to its dynamic nutrient microenvironment

**DOI:** 10.1038/s41419-020-2449-5

**Published:** 2020-04-20

**Authors:** Shiva Kant, Pravin Kesarwani, Antony Prabhu, Stewart F. Graham, Katie L. Buelow, Ichiro Nakano, Prakash Chinnaiyan

**Affiliations:** 1 Department of Radiation Oncology, Beaumont Health, Royal Oak, MI USA; 2 Department of Metabolomics and Obstetrics/Gynecology, Beaumont Research Institute, Beaumont Health, Royal Oak, MI USA; 3 Department of Neurosurgery, University of Alabama at Birmingham, Alabama, USA; 4 Oakland University William Beaumont School of Medicine, Royal Oak, MI USA

**Keywords:** Metabolomics, Cancer metabolism, CNS cancer

## Abstract

Despite advances in molecularly characterizing glioblastoma (GBM), metabolic alterations driving its aggressive phenotype are only beginning to be recognized. Integrative cross-platform analysis coupling global metabolomic and gene expression profiling on patient-derived glioma identified fatty acid β-oxidation (FAO) as a metabolic node in GBM. We determined that the biologic consequence of enhanced FAO is directly dependent upon tumor microenvironment. FAO serves as a metabolic cue to drive proliferation in a β-HB/GPR109A dependent autocrine manner in nutrient favorable conditions, while providing an efficient, alternate source of ATP only in nutrient unfavorable conditions. Rational combinatorial strategies designed to target these dynamic roles FAO plays in gliomagenesis resulted in necroptosis-mediated metabolic synthetic lethality in GBM. In summary, we identified FAO as a dominant metabolic node in GBM that provides metabolic plasticity, allowing these cells to adapt to their dynamic microenvironment. Combinatorial strategies designed to target these diverse roles FAO plays in gliomagenesis offers therapeutic potential in GBM.

## Introduction

The World Health Organization classifies glioma into Grades I–IV based on specific pathologic criteria that influence prognosis and clinical management. Grade I tumors are typically cured by surgical resection, whereas patients with Grade IV tumors, termed glioblastoma (GBM), have a median survival of less than 2 years despite aggressive multi-modality therapy^1^. Although considerable progress has been made in understanding the underlying biology of glioma from a genomic perspective, metabolic alterations that drive the aggressive phenotype of GBM are only beginning to be recognized. Similar to other tumors, GBM is known to be a highly glycolytic malignancy and accordingly, a majority of metabolism-based research has focused on this pathway^2^. Additional metabolic pathways that have received considerable attention in these rapidly proliferating tumors include glutamine metabolism^3,4^ and fatty acid synthesis (FAS)^5^. While LGG is typically a histologically homogeneous tumor, GBM is the archetypal example of a heterogeneous malignancy with a diverse tumor ecology that includes regions of hypoxia, necrosis, angiogenesis, and differential nutrient gradients within an individual tumor^6^. Therefore, dynamic metabolic reprogramming likely plays a central role during gliomagenesis, allowing these cells to adapt, survive, and proliferate in the diverse microenvironment implicit in this tumor. To provide a window into metabolic programs driving malignant transformation in glioma, we recently performed a comprehensive investigation comparing low grade astrocytoma (LGA) and GBM metabolism using global metabolomic profiling on patient-derived tumors^7^. Rather than FAS, these integrative analyses uncovered a highly coordinated, redundant metabolic program shifting from FAS to catabolism in GBM, with FA β-oxidation (FAO) emerging as a key metabolic node differentiating GBM from LGA.

A number of recent investigations have linked enhanced FAO, which represents a multistep process by which FAs are oxidized and broken down into carbon substrates, with tumorigenesis^8^. The NADH and FADH2 generated during β-oxidation and acetyl CoA oxidation in the TCA cycle provide the electron gradient required for ATP synthesis during oxidative phosphorylation. Although a majority of investigations have ascribed energy production as the biological consequence of enhanced FAO in cancer, the diverse roles FAO may play beyond ATP, including cataplerotic reactions that provide substrates for amino acids, nucleotide synthesis, and improved redox potential, are now being recognized^8–11^.

In this report, we define the metabolic phenotype of enhanced FAO in GBM, uncovering novel roles FAO plays in providing metabolic plasticity in these tumors; thereby, allowing GBM cells to accommodate to its dynamic microenvironment. This includes serving as a metabolic cue to drive proliferation in nutrient favorable conditions through a β-HB/GPR109A dependent manner, while serving as an efficient, alternate source of ATP only in nutrient unfavorable conditions. Additionally, we identify rational combinatorial strategies designed to target these dynamic roles FAO plays in gliomagenesis, resulting in metabolic synthetic lethality in GBM.

## Materials and methods

### Cell culture

Mesenchymal (MES83, MES326, and MES1027A) and proneural (PN19, PN84) GBM tumor-initiating cells were generated, obtained, authenticated and provided by Dr. Nakano^12^. Cells were cultured in DMEMF/12, GlutaMAX™ (Gibco-Thermofisher, Grand Island, NY) media supplemented with EGF (PeproTech, Rockey Hill, NJ), FGF (Prospec, Ness-Ziona, Israel), Heparin (STEMCELL Technologies Inc., Cambridge, MA), B-27 (Gibco, Grand Island, NY) with penicillin streptomycin as described^12^ and from hereinafter referred as regular /nutrient favorable media. Trypan blue dye exclusion was used to determine viable and dead cells.

### Reagents

FA free BSA, palmitate, linoleic acid, oligomycin, carbonyl cyanide-4-(trifluoromethoxy) phenylhydrazone (FCCP), rotenone, antimycin A, sodium citrate and beta hydroxybutyrate (β-HB) sodium salt were purchased from Sigma Aldrich (St. Louis, MO). Etomoxir was obtained from Sigma Aldrich (St. Louis, MO) or Cayman Chemicals (Ann Arbor, MI). 2-deoxyglucose (2DG) was obtained from Cayman Chemicals (Ann Arbor, MI).

### Metabolomic profiling and data analysis

Metabolomic profiling of patient-derived tumors and cell lines were performed as described previously^7^ and medium and long chain acylcarnitines were analyzed using MetaboAnalyst v.3.0^13^. Hierarchical clustering was performed with log-transformed normalized data using Euclidean distance and Ward scaling. Missing values were replaced by half of the minimum value. O[6]-methylguanine-DNA methyltransferase (MGMT) methylation and IDH1 status of tumors was performed as described previously^7^. Quantitative metabolomic analysis of acylcarnitines and amino acids was performed using the Absolute IDQ p180 kit (Biocrates Life Sciences AG, Innsbruck, Austria). Briefly, after treatment with etomoxir (40 µM, 24 h), cells were washed with chilled PBS and then lysed in 10 mM phosphate buffer and analyzed according to manufacturer instructions. Metabolites below the limit of detection (LOD) in all treatment conditions or were identified in less than two samples in a group were not reported.

### Cellular bioenergetics

FAO was measured by changes in oxygen consumption rate (OCR) following FAO inhibition or palmitate-induction as described^14^, using the Seahorse XF24 platform (Agilent, Santa Clara, CA). Briefly, to measure palmitate induction of FAO, cells were seeded overnight in substrate-limited DMEM media (Gibco, Thermofisher Life Technologies, Grand Island, NY), supplemented with 0.5 mM glucose, 1 mM glutamax, 0.5 mM l-carnitine, 0.1% B-27, EGF, FGF and heparin. Cells were then washed and re-seeded in FAO assay medium (111 mM NaCl, 4.7 mM KCl, 1.25 mM CaCl_2_, 2 mM MgSO_4_, and 1.2 mM NaH_2_PO_4_) supplemented with 2.5 mM glucose, 0.5 mM carnitine, and 5 mM HEPES pH 7.4 on Seahorse cell culture plates and incubated for 45 min in a non-CO_2_ incubator. BSA alone or BSA-conjugated palmitate (300 μM) was injected through the port. Data is represented as a difference in OCR between BSA-palmitate and BSA. FAO-dependent and independent mitochondrial OCR was measured in cells seeded on Seahorse cell culture plates in XF media (Agilent, Santa Clara, CA) supplemented with an equimolar concentration of glucose, glutamine and pyruvate as DMEMF/12 followed by treatment with etomoxir (40 μM; 45 min). The difference in basal OCR in etomoxir treated and non-treated cells was calculated from total mitochondrial OCR, measured using oligomycin (2 µM), carbonyl cyanide-4-(trifluoromethoxy) phenylhydrazone (FCCP) (1 µM), rotenone (0.5 µM), and antimycin (0.5 µM). Glycolysis was measured via extracellular acidification rate (ECAR). Briefly, cells were seeded in XF media supplemented with Glutamax (2.5 mM) and incubated for 45 min in a non-CO_2_ incubator. After basal ECAR, glucose (17.5 mM) was injected through the Seahorse port followed by the indicated concentration of 2DG. XF plates were pretreated with poly-d lysin, plated with 70,000 single cells, followed by brief centrifugation and incubation for 45 min before performing the assay. Lactate was measured in the cell culture supernatant using an l-lactate assay kit (Eton Biosciences, CA).

### ATP quantification

Cells were cultured in regular/nutrient favorable and nutrient restricted/deprived media (NaCl 116 mM, KCl 4.7 mM, NaHCO_3_ 25 mM, MgSO_4_ 1.2 mM, KH_2_PO_4_ 1.2 mM, CaCl_2_ 1.3 mM, equimolar linoleic acid, and 0.1× B-27, pH 7.4). After treatment with the described compounds at indicated time points, cells were collected and ATP was quantified with an ATP determination kit (Invitrogen, Grand Island, NY) according to the manufacturer instructions.

### Beta hydroxybutyrate quantification

Cellular β-HB was quantified using a colorimetric assay kit (Cayman Chemicals, Ann Arbor, MI). Briefly, to study palmitate dependent β-HB production in nutrient favorable condition, cells were first primed to oxidize exogenous FA by culturing overnight in substrate limited DMEM media. Cells were then washed, re-seeded in DMEM media supplemented with equimolar concentrations of glucose, pyruvate, and glutamine. Cells were then treated with BSA alone or BSA-conjugated palmitate (500 μM, 4 h). β-HB was also measured in cells cultured in regular and nutrient-restricted media followed by inhibiting FAO using etomoxir (40 μM; 8 h). After treatment, cells were collected, and samples were prepared and analyzed according to manufacturer instructions. Data were normalized using protein concentration. Extracellular β-HB was quantified in concentrated culture supernatant from cells grown in regular media treated with or without etomoxir (40 μM; 8 h) using a β-HB assay kit (Abcam Cambridge, MA) according to manufacturer instructions.

### Cyclic AMP quantification

Intracellular cyclic AMP was quantified by ELISA (Cayman, Ann Arbor, USA). Briefly, cells were treated in the indicated treatment conditions, collected, and cAMP samples were prepared. The assay was performed according to manufacturer instructions and protein concentration was used to normalize samples.

### Analysis of mitochondrial superoxide

MitoSOX™ Red Mitochondrial Superoxide Indicator (Invitrogen, Grand Island, NY) was used to quantify mitochondrial superoxide and performed according to manufacturer instructions. Briefly, after indicated treatment, cells were stained with MitoSoX Red (5 µM) for 10 min in HBSS media at 37 °C and fluorescence was measured using a SpectraMax Gemini™ EM Microplate Spectrofluorometer (Molecular Devices, San Jose, CA) as described^15^.

### Western blot

Histones were extracted using a histone extraction kit (Abcam Cambridge, MA) according to manufacturer protocol. To induce necroptosis and serve as a positive control, cells were treated with pan-caspase inhibitor Q*-*VD*-*OPh (25 μM) and BV6 (7.5 μM) for 30 min followed by incubation with TNF-α (50 ng/ml). Western blot was performed using methods previously described^16^. Antibodies against p27 (3686), RIP1 (4926), acetylated H3K9 (9649S), and H3K14 (7627S) were obtained from Cell Signaling Technology (Danvers, MA). Antibodies against phosphorylated MLKL (ab187091), MLKL (ab184718), and RIP3 (ab56164) were obtained from Abcam (Cambridge, MA). Antibody against H3 (06–755) was purchased from Upstate (Lake Placid, NY) and tubulin (CP-06) from Calbiochem (San Diego, CA). HRP conjugated secondary antibodies were obtained from Sigma Aldrich (St. Louis, MO).

### Gene expression analysis

Expression profiles and molecular subtyping of GBM (*n* = 56) was performed as described previously^7^. Differential gene expression in mesenchymal cells treated with etomoxir (40 µM) alone or with β-HB (0.5 mM) for 48 h were analyzed using Affymetrix Human U133A Plus 2.0 Arrays (Life Technologies Corporation Grand Island, NY). Total RNA from cells was isolated using an RNA extraction kit (Bio-Rad, CA). RNA quantification, integrity, and array analysis after hybridization and scaling were performed as discussed previously^7^. Robust multi-array average (RMA) normalization and differential expression analysis was performed using Affymetrix expression console and transcriptome analysis software. Differentially expressed genes having a linear 2-fold change and *p*-value < 0.05 was considered for further analysis. Microarray data is submitted to ArrayExpress (Accession no; E-MTAB-8017).

### siRNA transfection

siRNA targeting RIP1, p27, CPT1A, GPR43, and GPR109A or a non-targeting scrambled sequence was used for transient knockdown (Thermofisher, Grand Island, NY). Indicated cells were transfected with siRNAs using Lipofectamine 2000 (Thermofisher Life Technologies, Grand Island, NY) for 8 h in DMEM F-12 plain media without B-27 and cells were reseeded in DMEM-F-12 complete media.

### Real-time PCR

Total RNA was isolated from cells using an Aurum™ Total RNA Mini Kit (BioRad, Hercules, CA). cDNA was prepared from total RNA using an iScript cDNA Synthesis Kit (BioRad, Hercules, CA). Expression of genes was determined after 48 h. treatment with siRNA using advance SYBR green (Bio-rad, Hercules, CA) and ViiA 7 Real-Time PCR System (Applied Biosystems, Foster City, CA). Primer pairs used in quantitative real-time PCR experiments: GPR109A Forward 5′-ATGTTGGCTATGAACCGCCAG-3′ Reverse 5′-GCTGCTGTCCGATTGGAGA-3′, β-Actin Forward 5′-GGATCAGCAAGCAGGAGTATG-3′ Reverse 5′-AGAAAGGGTGTAACGCAACTAA-3′, GPR43 Forward 5′-CCGTGCAGTACAAGCTCTCC-3′ Reverse 5′ CTGCTCAGTCGTGTTCAAGTATT-3′, CPT1A Forward 5′- GCCTCGTATGTGAGGCAAAA-3′, Reverse 5′-TCATCAAGAAATGTCGCACG-3′

### Flow cytometric analysis of apoptotic cells

Apoptotic cells were evaluated after indicated treatments by staining with Annexin V (Invitrogen, Carlsbad, CA) and 7AAD (Calbiochem, San Diego, CA) according to manufacturer instructions. Samples were analyzed on a BD FACS Canto II flow cytometer (Becton Dickon; Mount View, CA). The analysis was performed on FlowJo V.10 Software (FlowJo, LLC; Ashland, OR).

### IRB approval for human samples

Glioma/GBM tissues were obtained from the Moffitt Cancer Center tissue core facility approved by the ethics committee of the Moffitt Cancer Center (MCC 16197). For the use of specimens, patients gave their written consent.

### Animal handling

All in vivo experiments were performed according to institutional guidelines and approved by the Institutional Animal Care and Use Committee of Beaumont Health. Orthotopic xenograft of MES83 tumors were established in female nu/nu mice (Charles River Laboratories, USA) using methods previously described^12^. The animals were not included in the analysis if cells were *back flowed*/leaked during orthotopic implant. Mice were imaged using MRI after 10–12 days of tumor implant. Kaplan–Meier curves were used to analyze survival. Animals were not randomized in this study. Mice were euthanized after reaching endpoint criteria, evaluated by the animal facility staffs who were blinded to the outcome of the study.

### Software use

3D structure of fatty acid was generated using DIY-molecules^17^.

### Statistical analysis

A log-rank test was used for survival analyses and Kaplan–Meier method was used to generate a survival plot. Datasets were tested for normal distribution using Kolmogorov*–*Smirnov test followed by one-way ANOVA with Tukey’s test (multiple groups) or unpaired, two-tailed Student’s *t*-tests (two groups), Mann*–*Whitney test (non-normal datasets) using Origin Pro 2019 software (Origin Lab Corporation, Northampton, MA). *p* value of less than 0.05 was considered significant. All experiments were performed in triplicates otherwise indicated. Data presented as individual data points or mean ± SD (standard deviation). The boxes represent interquartile range, median, and whisker denotes upper and lower limit.

## Results

### Enhanced FAO represents a metabolic phenotype in mesenchymal GBM

**Fig. 1:**
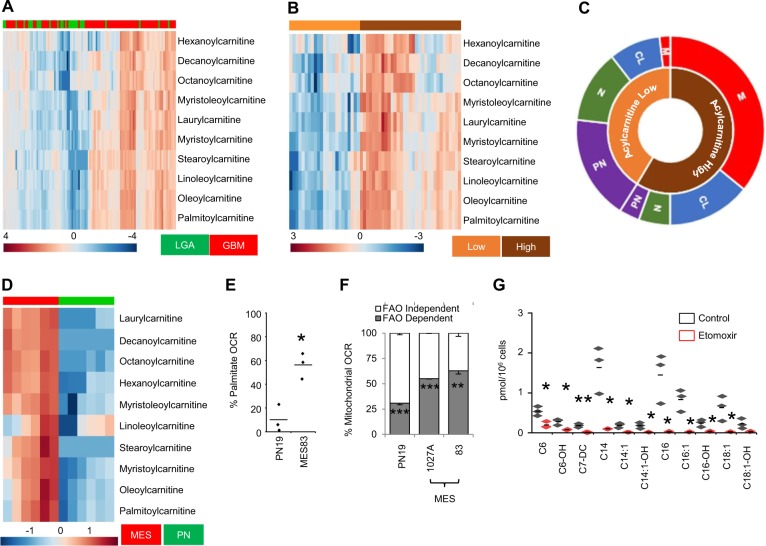
FAO related metabolites differentially accumulate in GBM. **a** Unsupervised clustering of patient-derived low-grade astrocytoma (LGA; *n* = 28) and glioblastoma (GBM; *n* = 80) demonstrates an accumulation of medium and long-chain acylcarnitines in GBM. **b** Heterogeneous clustering of acylcarnitines is observed in GBM (*n* = 56), demonstrating acylcarnitine “high” and “low” metabolic phenotypes. **c** The acylcarnitine “high” phenotype is enriched with mesenchymal (M) and classical (CL) subtypes, while neural (N) and proneural (PN) subtypes are enriched in the acylcarnitine “low” phenotype. **d** Unsupervised clustering of two patient-derived mesenchymal (MES) and two proneural (PN) GBM stem cell lines demonstrate an accumulation of medium and long-chain acylcarnitines (*n* = 3). **e** Cellular oxygen consumption rate (OCR) was measured in real time in PN and MES cells using the Seahorse platform (*n* = 3). Results represent percent increases in OCR following the addition of palmitate (300 µM). **f** Basal mitochondrial OCR was measured in stated cell lines treated with +/− etomoxir (40 µM; 45 min; *n* = 3). **g** Medium and long chain acylcarnitines were quantified in MES83 cells treated with +/− etomoxir (40 µM; 24 h; *n* = 3). Data represents mean ± SD (F) and line between the data points represents mean. *p < 0.05; **p < 0.005; ***p < 0.0005.

To define specific metabolic programs contributing towards gliomagenesis, we performed global metabolomic profiling on patient-derived low-grade astrocytoma (LGA; *n* = 28) and GBM (*n* = 80) using a metabolic library consisting of more than 3000 purified standards^7^. Through these studies, we demonstrated considerable metabolic reprogramming associated with gliomagenesis, with aberrant lipid metabolism representing a dominant node in GBM. Specifically, all the acylcarnitines identified were elevated in GBM when compared to LGA (differential abundance score = 1), with some demonstrating over 20-fold increases. We therefore explored this metabolic phenotype in further detail. Although, as expected, unsupervised clustering of acylcarnitines separated LGA and GBM, considerable heterogeneity was observed within GBM (Fig. 1a). To begin to understand factors contributing to this heterogeneity, we focused exclusively on GBM samples, which, following unsupervised clustering, demonstrated two clear phenotypes we defined as acylcarnitine “high” and “low” (Fig. 1b). To determine if the observed heterogeneity was a direct consequence of established molecular subtypes in GBM, we performed cross-platform analyses using RNA (expression profiling) and DNA (IDH1 mutation and MGMT promoter methylation) isolated from matched tumor tissue. Interestingly, we demonstrated that MGMT promoter methylation and IDH1 mutation, two of the strongest prognostic factors in GBM^18,19^ were evenly distributed among subtypes (Supplementary Fig. S1). Next, using expression profiles generated from these matched samples, tumors were classified according to their molecular subtype: mesenchymal (MES), proneural (PN), classical (C), or neural (N)^20^. This resulted in clear clustering, with MES and PN subtypes nearly exclusively present in FAO “high” and “low” subtypes, respectively (Fig. 1c).

As the FAO metabolic phenotype was enriched in MES tumors, we continued investigations to explore and understand the function of FAO in well-characterized, subtype-specific, preclinical models recapitulating MES and PN subtypes of GBM^6,12,16^. Global metabolomic profiling of these cell lines revealed a similar pattern of accumulation of FAO-related metabolites in aggressive MES when compared to PN lines, with several metabolites demonstrating 50–100-fold increases (Fig. 1d). Because metabolomics only provides a static picture of intermediary metabolism, the accumulation of FAO metabolites might reflect increased activation and/or utilization of this metabolic pathway or, conversely, dysfunction or saturation of a downstream enzyme. Therefore, we determined whether FAO was functionally active in MES cells. As an initial investigation, we evaluated oxygen consumption rate (OCR) in MES cells after addition of exogenous BSA-conjugated palmitate using the Seahorse XF platform^14,21^. The addition of palmitate resulted in a robust increase in OCR in MES cells when compared to PN, suggesting that the molecular machinery involved in FAO is intact in these lines (Fig. 1e). Next, we determined the relative contribution of FAO in overall cellular mitochondrial respiration. For these studies, FAO-dependent respiration was defined as a difference in baseline OCR in cells treated with or without the FAO/CPT1 inhibitor etomoxir (Supplementary Fig. S2A). Surprisingly, these studies demonstrated that an overwhelming majority of mitochondrial respiration in MES is a direct consequence of FAO. Nearly 60% of mitochondrial respiration of MES was dependent on FAO, while only ~25% was observed in PN cells (Fig. 1f); thereby, functionally recapitulating metabolomic findings generated from clinical samples. We next sought to determine if inhibiting FAO could revert the FAO “high” phenotype in MES cells. Targeted metabolomics demonstrated a decrease in accumulation of both medium and long-chain acylcarnitines following treatment with etomoxir (Fig. 1g). As short chain acylcarnitines are able to diffuse through the mitochondrial membrane in a CPT1-independent manner^22^, as expected, no change in these metabolites was observed (Supplementary Fig. S2B).

### The biologic consequence of FAO is dependent on microenvironment

**Fig. 2:**
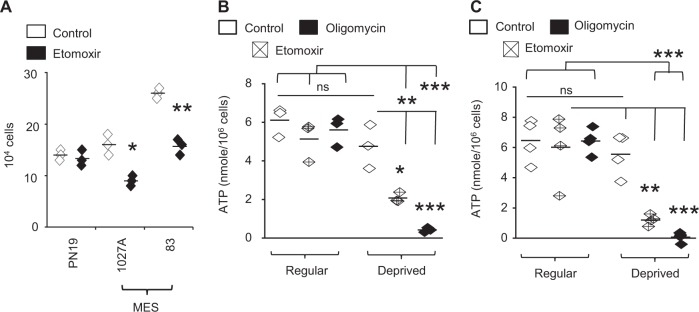
FAO regulates mesenchymal cell proliferation. **a** PN and MES cells were treated with +/− etomoxir (40 µM; 48 h) and live cells were counted using trypan blue (*n* = 3). ATP was measured in **b** MES83 (*n* = 3) and **c** 1027 A (*n* = 4) cells plated in regular and nutrient-deprived media treated with +/− etomoxir (40 µM; 4 h) or oligomycin (2 µM; 1 h). Line between the data points represents mean. *p < 0.05; **p < 0.005; ***p < 0.0005.

We extended investigations to begin to understand the biologic consequence of enhanced FAO in GBM, with initial studies evaluating its potential to serve as a therapeutic target. As expected, etomoxir demonstrated differential activity in MES and PN, with anti-proliferative effects noted in MES lines, (Fig. 2a). As the oxidation of FA yields significantly more energy per carbon atom than carbohydrates^8^, a likely biologic advantage offered by enhanced FAO in GBM would be to serve as a unique energy source. Unexpectedly, ATP levels remained unchanged after FAO inhibition in MES cell lines (Fig. 2b, c). This was not due to a compensatory increase in glycolysis after FAO inhibition (Supplementary Fig. S2C). Similarly, treating cells with the established ATP synthase inhibitor oligomycin also did not influence ATP levels in these conditions (Fig. 2b, c), confirming that although considerable FAO-dependent mitochondrial respiration occurs in MES, it does not result in ATP synthesis. Based on these findings, we hypothesized that FAO may play different roles in gliomagenesis dependent on the availability of nutrients in the microenvironment. As an initial investigation, we determined if FAO might contribute to ATP synthesis in nutrient-deprived conditions. Interestingly, MES cells demonstrated the capacity to maintain ATP levels even when switching culture media from nutrient favorable to deprived conditions. This was dependent on FAO, as treating cells in these conditions with etomoxir or oligomycin resulted in near complete inhibition of ATP synthesis (Fig. 2b, c). Collectively, these findings suggest that FAO may serve multiple functions in gliomagenesis depending on the energetic state and/or tumor microenvironment. We therefore went on to evaluate the possibility for these differential roles in further detail.

### FAO leads to an accumulation of β-HB in nutrient favorable conditions

**Fig. 3:**
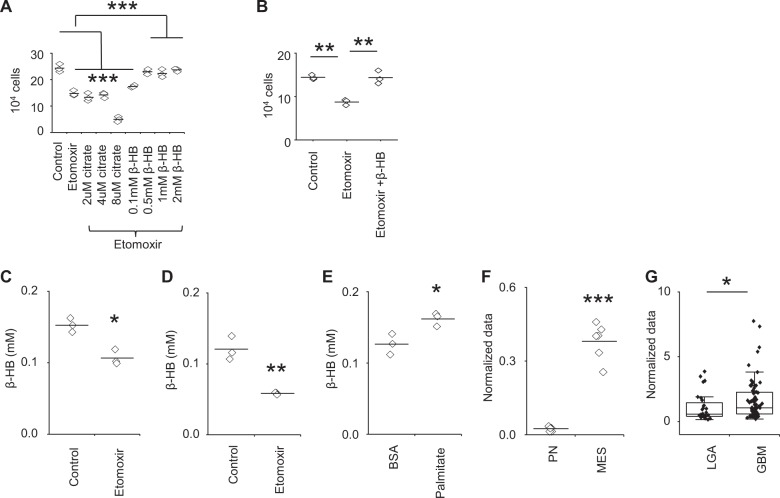
MES cells produce β-HB in nutrient-rich conditions. **a** MES83 cells were treated with +/− etomoxir (40 µM) or in combination with indicated concentration of citrate or β-HB (*n* = 3). Viable cells were counted using trypan blue after 48 h. **b** 1027 A cells were treated with +/− etomoxir (40 µM) or in combination with β-HB (0.5 mM) (*n* = 3). Viable cells counted using trypan blue after 48 h. Cellular β-HB was measured in **c** MES83 and **d** 1027 A cells treated with +/− etomoxir (40 µM; 8 h; *n* = 3). **e** Cellular β-HB was measured in MES83 cells treated with BSA and BSA:palmitate (500 µM; 4 h; *n* = 3). Concentration of β-HB in **f** two patient-derived mesenchymal (MES) and 2 proneural (PN) GBM stem cell lines (*n* = 3) and **g** in patient low-grade astrocytoma (LGA; *n* = 28) and glioblastoma (GBM; *n* = 80, data truncated at 10). The boxes represent interquartile range, median and whisker denotes upper and lower limit. Line between the data points represents mean. *p < 0.05; **p < 0.005; ***p < 0.0005.

We next sought to determine the biologic consequence of FAO in MES cells cultured in standard, nutrient-favorable conditions. As the metabolic “cost” of fueling the electron transport chain without yielding ATP (i.e., oxidation without phosphorylation or endogenous uncoupling) is seemingly high, requiring oxygen and resulting in free radical generation^23^, we hypothesized that metabolic intermediaries of FAO contribute towards vital aspects of gliomagenesis. We therefore explored other potential fates of the resultant acetyl CoA generated by FAO to determine the biologic advantage conferred by this adaptation. The first branching point of acetyl CoA is metabolism towards citrate or ketogenesis. The resultant citrate, which forms after acetyl CoA combines with oxaloacetate, represents the most common route, which can then be further metabolized through a variety of pathways, including proceeding through the TCA cycle and generating ATP and/or other key amino acids implicated in tumorigenesis, or exported out of the mitochondria to be utilized for FA synthesis^8^. Alternately, through a pathway typically ascribed to the liver, acetyl CoA can be metabolized towards ketogenesis, which is secreted in the blood and able to provide tissues an alternative substrate to glucose during prolonged starvation^24^. As an initial investigation to understand the biologic consequence of FAO in GBM in nutrient favorable conditions, we performed metabolomic analysis on a focused panel of amino acids, which did not demonstrate any changes following FAO inhibition (Supplementary Fig. S3A). Next, as both citrate and β-HB are taken up by cells^25,26^, we determined if the anti-proliferative effects of FAO inhibition could be rescued by exogenous addition of these metabolites. β-HB, but not citrate, demonstrated the capacity to rescue MES cells from the anti-proliferative effects of the CPT1 inhibitor etomoxir (Fig. 3a, b), which was further validated through molecular silencing of this enzyme (Supplementary Fig. S4A, B). Further, we confirmed that inhibiting FAO by etomoxir (Fig. 3c, d) or culturing cells in nutrient-deprived conditions (Supplementary Fig. S3B) led to a decrease in β-HB, and adding exogenous palmitate led to an accumulation of β-HB (Fig. 3e). Additionally, high levels of baseline β-HB was observed in MES when compared to PN cells (Fig. 3f), and importantly, these findings were recapitulated in clinical samples, with increased levels of β-HB observed in GBM (Fig. 3g). Collectively, these findings support the contributory role of β-HB in FAO-mediated proliferation of GBM.

### β-HB serves as a metabolic cue to drive FAO-mediated proliferation in a GPR109A/cAMP dependent manner

We next sought to define the mechanism of FAO/β-HB-mediated proliferation in GBM in the context of nutrient favorable conditions. As an initial investigation, we confirmed exogenous β-HB, which is able to rescue cells from anti-proliferative effects of etomoxir, is not further metabolized in the mitochondria (Supplementary Fig. S5). Beyond its role as a source of energy, recent investigations have demonstrated β-HB to serve as a dynamic signaling molecule that has the capacity to modulate cellular function at multiple levels^27^. We therefore went on to evaluate direct roles FAO-derived β-HB may play in proliferation. As β-HB was recently shown to serve as an endogenous HDAC inhibitor^26^, we tested the hypothesis that FAO-mediated accumulation of β-HB could activate transcriptional programs driving cellular proliferation. Although decreased acetylation of histone H3 was observed following FAO inhibition, which was reversed by the addition of exogenous β-HB (Supplementary Fig. S6A, B), subsequent transcriptional profiling did not reveal robust changes in gene expression following etomoxir that could explain observed changes in proliferation (Supplementary Table S1; Fig. S6C, D).

**Fig. 4:**
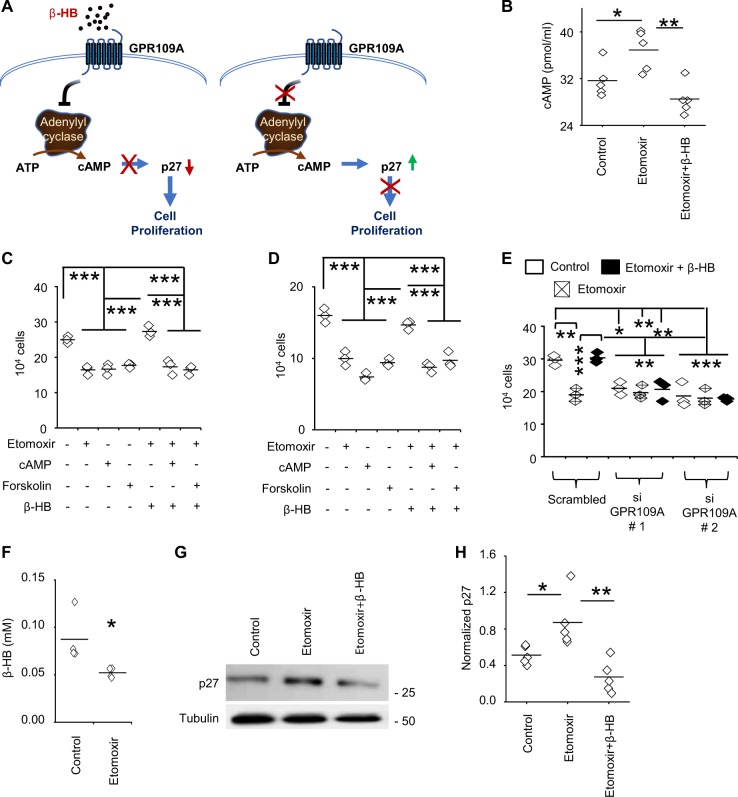
β-HB regulates cellular proliferation in a GPR109A/cAMP dependent manner. **a** Schematic representation of the β-HB/GPR109A signaling pathway. **b** Cellular cAMP was measured in MES83 cells treated with +/− etomoxir (8 h, 40 µM) alone and in combination with β-HB (0.5 mM; *n* = 5). **c** MES83 and **d** 1027 A cells were pretreated with +/− etomoxir (40 µM) and β-HB (0.5 mM) for 30 min followed by treatment with forskolin (40 μM) or 8-Br-cAMP (0.75 mM; *n* = 3). Viable cells were counted using trypan blue after 48 h. **e** MES83 cells were pretreated with GPR109A or scrambled siRNA (48 h; *n* = 3) and then treated in the described conditions. Viable cells were counted using trypan blue after 48 h. **f** β-HB was measured in culture supernatant of MES83 cells treated +/− etomoxir (40 µM; 8 h; *n* = 4). p27 expression was analyzed by **g** western blot in MES83 cells (*n* = 5) treated with +/− etomoxir (40 μM) alone or in combination with β-HB (0.5 mM) for 48 h and **h** expression was quantified by densitometry. Line between the data points represents mean. *p < 0.05; **p < 0.005; ***p < 0.0005.

G-protein coupled receptors (GPRs), which have multiple roles in regulating metabolism, have also recently been identified to serve as a molecular target of β-HB^27^. One example is GPR109A (HCAR2)^27,28^, whose activation has been shown to decrease levels of cAMP through inhibition of adenylyl cyclase^29^, and therefore, has potential implications in cellular proliferation (Fig. 4a)^30–32^. As an initial investigation, we confirmed FAO inhibition with etomoxir led to increased levels of cAMP, which were reversed by β-HB (Fig. 4b). Continuing to use proliferation as a functional readout, we went on to delineate the potential relevance of this signaling axis on β-HB-mediated proliferation in further detail by activating and/or inhibiting its downstream signaling intermediaries. The addition of exogenous cAMP and the adenylyl cyclase activator forskolin had similar anti-proliferative effects as etomoxir, which importantly, was not rescued by the addition of upstream β-HB (Fig. 4c, d). Knockdown of GPR109A using siRNA (Supplementary Fig. S7A) demonstrated similar anti-proliferative effects as FAO inhibition, was not further influenced by etomoxir, and was no longer rescued by β-HB (Fig. 4e), validating GPR109A as a primary target of β-HB-mediated cellular proliferation in GBM. Conversely, knockdown of GPR43, which represents an alternate GPR and putative target of β-HB, did not influence cellular proliferation or the ability of this metabolite to rescue cells from FAO mediated anti-proliferation (Supplementary Fig. S7B, C), further supporting the specific role GPR109A plays in this signaling pathway. As GPR109A represents an extracellular receptor, to further confirm our working model, we validated autocrine signaling by demonstrating a reduction of extracellular levels of β-HB following FAO inhibition (Fig. 4f). Next, we extended these findings to determine molecular mediators of cell cycle arrest modulated by the β-HB/GPR109A/cAMP axis. Consistent with proliferation studies, etomoxir led to robust expression of the cyclin-dependent kinase inhibitor p27, which was reverted with β-HB (Fig. 4g, h). These findings remained consistent when modulating downstream mediators of this autocrine axis, including treating cells with forskolin and siRNA knockdown of both GPR109A and p27 (Supplementary Fig. S8A–G). Collectively, these findings validate FAO-generated β-HB serves as a metabolic cue in nutrient favorable conditions, stimulating autocrine proliferation in a GPR109A/cAMP-dependent manner.

### FAO represents a metabolic vulnerability in glucose-deprived conditions

**Fig. 5:**
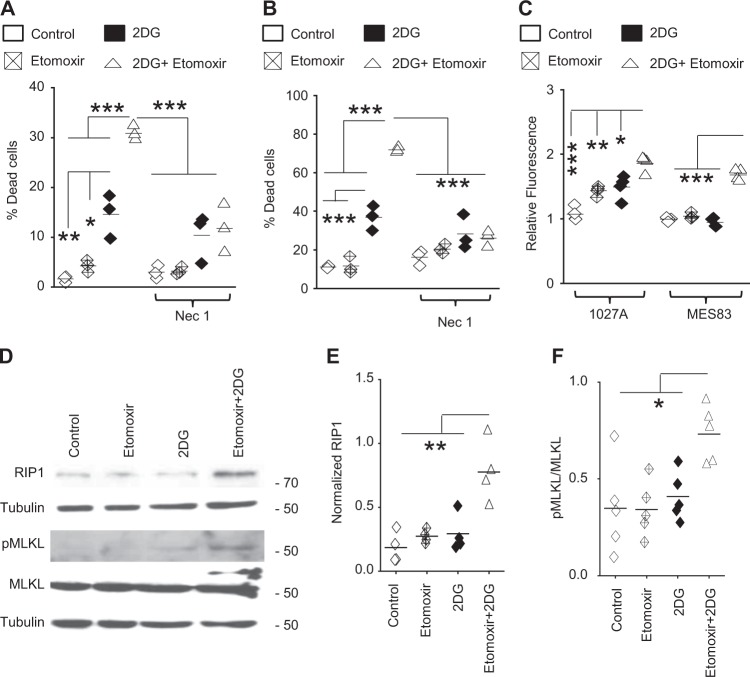
Dual inhibition of FAO and glycolysis elicits necroptosis-mediated metabolic synthetic lethality in MES cells in vitro. **a** MES83 and **b** 1027 A cells were +/– pretreated with necrostatin-1 (Nec-1; 100 µM, 30 min) and then treated with +/− etomoxir (40 µM), 2DG (35 mM) or the combination (*n* = 3). Non-viable cells were counted with trypan blue after 48 h. **c** Mitochondrial superoxide was evaluated with MitoSOX Red after 48 h. treatment with +/− etomoxir (40 µM), 2DG (35 mM) alone or in combination (*n* = 4). Expression of molecules involved in necroptosis was analyzed by western blot (**d**) after 48 hr treatment with indicated inhibitors. Normalized expression of **e** RIP1 (*n* = 4) and **f** pMLKL (*n* = 5) was quantified by densitometry. Line between the data points represents mean. *p < 0.05; **p < 0.005; ***p < 0.0005.

The above findings demonstrate that MES cells utilize FAO to serve as a metabolic cue to signal cellular proliferation in an autocrine manner in nutrient favorable conditions, and accordingly, inhibiting FAO alone only demonstrated modest inhibition of proliferation in these cells. However, functionally, we have shown these highly glycolytic cells rely upon FAO to serve as an important source of ATP in nutrient-restricted conditions. Therefore, we went on to test the hypothesis that dual targeting of FAO and glycolysis would lead to energetic stress, contributing towards metabolic synthetic lethality in GBM. For these studies, we used the glycolysis inhibitor 2-DG after determining the minimum dose required to inhibit glycolysis (35 mM; Supplementary Fig. S9A). Consistent with previous findings, FAO inhibition alone led to a modest inhibition of MES proliferation and minimum cytotoxicity. Although the glycolysis inhibitor 2-DG did elicit modest cytotoxicity, dual targeting of FAO and glycolysis resulted in robust synergistic cell death (Fig. 5a, b) and energetic stress, as determined by superoxide production quantified by MitoSox (Fig. 5c)^33^, suggesting the potential for this combination to serve as a rationale treatment strategy in GBM. Next, we explored potential mechanisms contributing to the observed cell death. After confirming a non-apoptotic mechanism (Supplementary Fig. S9B), we evaluated necroptosis, which represents a mode of cell death that has been previously attributed to energetic stress^34,35^. Interestingly, the robust cell death elicited by the addition of 2-DG with etomoxir (Fig. 5a, b) or CPT1A knockdown (Supplementary Fig. S9C) was mitigated with the necroptosis inhibitor necrostatin-1. Cells treated with the etomoxir and 2-DG combination demonstrated increased expression of the necroptosis markers RIP1 and phospho-MLKL (Fig. 5d–f). Although no changes in RIP3 expression was observed in our model (Supplementary Fig. S10A–D), a similar observation has been reported previously^36^, suggesting alternate mechanisms by which necroptosis may be initiated. Knockdown of RIP1 using siRNA mitigated the increased cytotoxicity observed in cells treated with the etomoxir and 2-DG combination, further implicating RIP1 signaling as an initiator of the observed necroptosis-mediated cell death (Supplementary Fig. S10E–G).

**Fig. 6:**
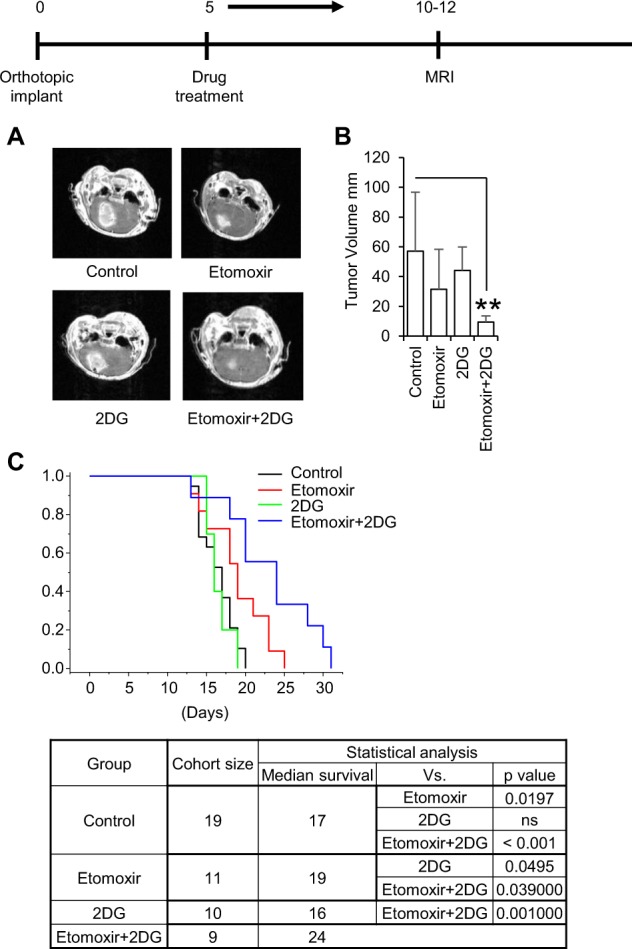
Dual inhibition of FAO and glycolysis improves survival in a orthotopic GBM mouse model. Etomoxir (40 mg/kg) and/or 2-DG (500 mg/kg) were administered via intraperitoneal (i.p.) injection 5 days a week. **a**, **b** Mice were imaged and tumors measured using MRI 10–12 days following MES83 tumor implant. Data represents mean ± SD. **p < 0.005. **c** Kaplan–Meier survival plot and statistical analysis in mice treated with the indicated agents.

Based on the promising results involving the combination of FAO and glycolysis inhibitors in GBM, we extended our work in vivo using an orthotopic model. The combination of etomoxir and 2-DG was well tolerated in mice (Supplementary Fig. S11A). MR imaging performed 5–7 days following treatment demonstrated clear anti-tumor activity with the etomoxir and 2-DG combination (Fig. 6a, b). When evaluated for survival, consistent with in vitro findings, etomoxir demonstrated modest anti-tumor activity, which was reflected by an increase in p27 expression in vivo (Supplemental Fig. S11B, C), although, 2-DG, at the dose used in these studies, did not demonstrate activity when given as a single agent (Fig. 6c). The combination of etomoxir and 2-DG resulted in a significant improvement in survival, supporting the potential for this dual-targeted metabolic strategy to be extended clinically.

## Discussion

Alterations in lipid metabolism have been described to play a contributory role in tumorigenesis in a number of malignancies, however, an overwhelming majority of these studies have focused on de novo FAS^37–39^. Recent discoveries have placed a new emphasis on FAO as an important metabolic node in cancer^8^. For example, heterogeneity in fuel utilization was identified in B-cell lymphoma, with FAO serving as a major energy source in the “OxPhos” subtype through BCR independent signaling^21^ and Nieman et al. identified a unique mechanism by which adipocytes provide energy for omental ovarian cancer metastases through FAO^40^. FAO has also been implicated in breast cancer pathogenesis, contributing to growth in a PML-dependent manner^41^ and stem cell renewal and therapeutic resistance^42^. The complex, biologic consequence of FAO goes well beyond solely serving as an energy source^8^, including its capacity to provide substrates for NADPH production to modulate redox stress^9^, pyrimidines precursors in endothelial cells^10^, and metabolic intermediates to promote stemness in hematopoietic cells^43^. Accordingly, the potential for this metabolic node to serve as a therapeutic target has gained recent attention^8,44–46^.

Although several studies have suggested metabolic programming leading to activation of FAO is involved in tumorigenesis, including in glioma^47^, a majority of these studies have relied upon aberrant expression of genes associated with this pathway. Our study, which utilized global metabolomic profiles generated from over 100 patient samples, is amongst the first to establish FAO as a clear metabolic node in GBM. This metabolic phenotype was defined by a robust accumulation of acylcarnitines that was both biologically and functionally recapitulated in preclinical models. This is consistent with our previous integrative analysis that demonstrated a key metabolic alteration associated with malignant transformation in glioma involves a shift from FAS to FAO^7^. Further, by coupling metabolomics with genomic profiles, these findings provide a previously undescribed window into intratumoral metabolic heterogeneity in GBM. For example, the observed FAO phenotype was specific to the mesenchymal and classical subtypes of GBM, which we previously have shown to be reflective of the perinecrotic/core region of an individual tumor^6^. These findings support the notion that dynamic metabolic reprogramming plays a contributory role in gliomagenesis, allowing these cells to adapt, survive, and proliferate in the diverse microenvironment implicit in this tumor. Further investigations designed to directly examine regional differences of FAO in GBM are warranted and may provide insight into novel combinatorial approaches to target this tumor’s metabolically diverse tumor ecology.

**Fig. 7:**
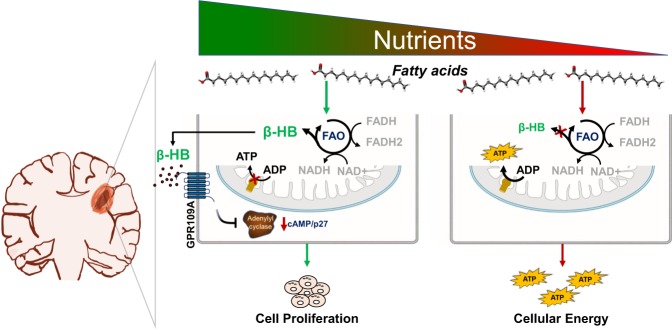
Diagram summarizing the dynamic roles FAO plays in GBM based on a given microenvironment. In nutrient favorable conditions (left), glioblastoma cells utilize FAO to stimulate proliferation through the β-HB/GPR109A/cAMP signaling axis. In nutrient unfavorable conditions (right), glioblastoma cells are able to utilize FAO to generate ATP to maintain survival.

Our findings provide a previously undescribed insight into the dynamic role FAO plays in maintaining metabolic plasticity in GBM. Specifically, we demonstrate that the biologic consequence of enhanced FAO is directly influenced by nutrient availability in a given microenvironment. Although often reflexively attributed to serving as a supplementary energy source, we demonstrate the sole reliance of these rapidly proliferating GBM cells on glycolysis as the primary source of ATP in standard culture conditions. This was further validated following treatment with the ATP synthase inhibitor oligomycin, which surprisingly, resulted in no net reduction in cellular ATP in GBM cells cultured in standard conditions. Through a series of investigations, we went on to discover that FAO serves as a metabolic cue that drives proliferation in nutrient favorable conditions in an autocrine manner through a β-HB/GPR109A/cAMP mediated-axis (Fig. 7). This is consistent with recent reports attributing β-HB with many dynamic roles in modulating disease and its capacity to serve as a link between the microenvironment and cellular function^48^. Interestingly, recent studies have demonstrated both anti-cancer and pro-cancer properties of these biochemical intermediates^49^. Specifically, β-HB is a measurable end metabolic consequence of a ketogenic diet (KD), whose potential to serve as an anti-cancer therapy remains an active area of investigation, particularly in glioma, and preliminary studies have suggested anti-cancer properties of this metabolite in vitro^50^. On the contrary, reports have also demonstrated that ketone bodies can contribute to tumor growth and metastases^49,51–53^. One particularly notable paper has linked ketogenesis with proliferation in BRAF mutant melanoma^54^, supporting some of our findings. Therefore, further studies designed to understand the metabolic interplay between FAO, glycolysis, and intratumoral metabolic changes resulting from a KD are warranted.

Another key discovery uncovered from this work was the dynamic and intimate relationship between FAO and glycolysis in gliomagenesis. The considerable metabolic “cost” required to maintain FAO suggests that the resulting metabolic plasticity it affords appears critical for these cells to accommodate and maintain both survival and proliferation in the dynamic microenvironment of GBM. In addition to providing a unique insight into metabolic heterogeneity in GBM, these findings have clear clinical implications. Specifically, glycolysis and FAO are both being individually examined as therapeutic targets in GBM. However, our findings suggest that targeting FAO alone in GBM would only result in modest anti-proliferative activity. Further, GBM would likely acquire resistance to targeting glycolysis alone, based on these cells’ capacity to efficiently reprogram their metabolism and switch to FAO to provide ATP required for continued survival. Combinatorial strategies targeting both FAO and glycolysis led to metabolic synthetic lethality in vitro and was well tolerated in vivo, leading to improved survival. Therefore, continued studies designed to identify tumor-specific mediators of both FAO and glycolysis and evaluating them in combination may have clinical implications in GBM therapy.

In summary, we identified FAO as a dominant metabolic node in GBM that provides metabolic plasticity, allowing these cells to adapt to their dynamic microenvironment. Combinatorial strategies designed target both FAO and glycolysis led to metabolic synthetic lethality in GBM cells, offering a novel therapeutic strategy in this otherwise invariably fatal malignancy.

## Supplementary information

Legends of supplemental figures

Supplementary Fig. 1

Supplementary Fig. 2

Supplementary Fig. 3

Supplementary Fig. 4

Supplementary Fig. 5

Supplementary Fig. 6

Supplementary Fig. 7

Supplementary Fig. 8

Supplementary Fig. 9

Supplementary Fig. 10

Supplementary Fig. 11

Supplementary Table

## References

[1] Ostrom QT (2018). CBTRUS statistical report: primary brain and other central nervous system tumors diagnosed in the United States in 2011–2015. Neuro-Oncology.

[2] Agnihotri S, Zadeh G (2016). Metabolic reprogramming in glioblastoma: the influence of cancer metabolism on epigenetics and unanswered questions. Neuro-Oncology.

[3] Tanaka K (2015). Compensatory glutamine metabolism promotes glioblastoma resistance to mTOR inhibitor treatment. J. Clin. Investig..

[4] DeBerardinis RJ (2007). Beyond aerobic glycolysis: transformed cells can engage in glutamine metabolism that exceeds the requirement for protein and nucleotide synthesis. Proc. Natl Acad. Sci. USA.

[5] Guo D (2009). EGFR signaling through an Akt-SREBP-1-dependent, rapamycin-resistant pathway sensitizes glioblastomas to antilipogenic therapy. Sci. Signal..

[6] Prabhu A, Kesarwani P, Kant S, Graham SF, Chinnaiyan P (2017). Histologically defined intratumoral sequencing uncovers evolutionary cues into conserved molecular events driving gliomagenesis. Neuro-Oncology.

[7] Prabhu, A. H. et al. Integrative cross-platform analyses identify enhanced heterotrophy as a metabolic hallmark in glioblastoma. *Neuro-Oncology*10.1093/neuonc/noy185 (2018). 10.1093/neuonc/noy185 PMC6380409 30476237

[8] Carracedo A, Cantley LC, Pandolfi PP (2013). Cancer metabolism: fatty acid oxidation in the limelight. Nat. Rev. Cancer.

[9] Jeon SM, Chandel NS, Hay N (2012). AMPK regulates NADPH homeostasis to promote tumour cell survival during energy stress. Nature.

[10] Schoors S (2015). Fatty acid carbon is essential for dNTP synthesis in endothelial cells. Nature.

[11] Randall, E. C. et al. Localized metabolomic gradients in patient-derived xenograft models of glioblastoma. *Cancer Res*. 10.1158/0008-5472.CAN-19-0638 (2019). 10.1158/0008-5472.CAN-19-0638 PMC7073296 31767628

[12] Mao P (2013). Mesenchymal glioma stem cells are maintained by activated glycolytic metabolism involving aldehyde dehydrogenase 1A3. Proc. Natl Acad. Sci. USA.

[13] Xia J, Sinelnikov IV, Han B, Wishart DS (2015). MetaboAnalyst 3.0–making metabolomics more meaningful. Nucleic Acids Res..

[14] Wang D, Green MF, McDonnell E, Hirschey MD (2013). Oxygen flux analysis to understand the biological function of sirtuins. Methods Mol. Biol..

[15] Wojtala A (2014). Methods to monitor ROS production by fluorescence microscopy and fluorometry. Methods Enzymol..

[16] Prabhu A (2015). Ras-mediated modulation of pyruvate dehydrogenase activity regulates mitochondrial reserve capacity and contributes to glioblastoma tumorigenesis. Neuro Oncol..

[17] Bienfait B, Ertl P (2013). JSME: a free molecule editor in JavaScript. J. Cheminform..

[18] Hegi ME (2005). MGMT gene silencing and benefit from temozolomide in glioblastoma. N. Engl. J. Med..

[19] Yan H (2009). IDH1 and IDH2 mutations in gliomas. N. Engl. J. Med..

[20] Verhaak RG (2010). Integrated genomic analysis identifies clinically relevant subtypes of glioblastoma characterized by abnormalities in PDGFRA, IDH1, EGFR, and NF1. Cancer Cell.

[21] Caro P (2012). Metabolic signatures uncover distinct targets in molecular subsets of diffuse large B cell lymphoma. Cancer Cell.

[22] Qu Q, Zeng F, Liu X, Wang QJ, Deng F (2016). Fatty acid oxidation and carnitine palmitoyltransferase I: emerging therapeutic targets in cancer. Cell Death Dis..

[23] Weinberg SE, Chandel NS (2015). Targeting mitochondria metabolism for cancer therapy. Nat. Chem. Biol..

[24] Puchalska P, Crawford PA (2017). Multi-dimensional roles of ketone bodies in fuel metabolism, signaling, and therapeutics. Cell Metab..

[25] Gameiro PA (2013). In vivo HIF-mediated reductive carboxylation is regulated by citrate levels and sensitizes VHL-deficient cells to glutamine deprivation. Cell Metab..

[26] Shimazu T (2013). Suppression of oxidative stress by beta-hydroxybutyrate, an endogenous histone deacetylase inhibitor. Science.

[27] Newman JC, Verdin E (2014). Ketone bodies as signaling metabolites. Trends Endocrinol. Metab..

[28] Taggart AK (2005). (D)-beta-Hydroxybutyrate inhibits adipocyte lipolysis via the nicotinic acid receptor PUMA-G. J. Biol. Chem..

[29] Kostylina G, Simon D, Fey MF, Yousefi S, Simon HU (2008). Neutrophil apoptosis mediated by nicotinic acid receptors (GPR109A). Cell Death Differ..

[30] Indolfi C (1997). Activation of cAMP-PKA signaling in vivo inhibits smooth muscle cell proliferation induced by vascular injury. Nat. Med..

[31] Schmitt JM, Stork PJ (2001). Cyclic AMP-mediated inhibition of cell growth requires the small G protein Rap1. Mol. Cell. Biol..

[32] Desdouets C (1995). Cell cycle regulation of cyclin A gene expression by the cyclic AMP-responsive transcription factors CREB and CREM. Mol. Cell. Biol..

[33] Abramov AY, Scorziello A, Duchen MR (2007). Three distinct mechanisms generate oxygen free radicals in neurons and contribute to cell death during anoxia and reoxygenation. J. Neurosci..

[34] Eguchi Y, Shimizu S, Tsujimoto Y (1997). Intracellular ATP levels determine cell death fate by apoptosis or necrosis. Cancer Res..

[35] Skulachev VP (2006). Bioenergetic aspects of apoptosis, necrosis and mitoptosis. Apoptosis.

[36] Caccamo A (2017). Necroptosis activation in Alzheimer’s disease. Nat. Neurosci..

[37] Currie E, Schulze A, Zechner R, Walther TC, Farese RV (2013). Cellular fatty acid metabolism and cancer. Cell Metab..

[38] Gabitova L, Gorin A, Astsaturov I (2014). Molecular pathways: sterols and receptor signaling in cancer. Clin. Cancer Res..

[39] Menendez JA, Lupu R (2007). Fatty acid synthase and the lipogenic phenotype in cancer pathogenesis. Nat. Rev. Cancer.

[40] Nieman KM (2011). Adipocytes promote ovarian cancer metastasis and provide energy for rapid tumor growth. Nat. Med..

[41] Carracedo A (2012). A metabolic prosurvival role for PML in breast cancer. J. Clin. Investig..

[42] Wang T (2018). JAK/STAT3-regulated fatty acid beta-oxidation is critical for breast cancer stem cell self-renewal and chemoresistance. Cell Metab..

[43] Ito K (2012). A PML-PPAR-delta pathway for fatty acid oxidation regulates hematopoietic stem cell maintenance. Nat. Med..

[44] Samudio I (2010). Pharmacologic inhibition of fatty acid oxidation sensitizes human leukemia cells to apoptosis induction. J. Clin. Investig..

[45] Grande S (2018). Metabolic heterogeneity evidenced by MRS among patient-derived glioblastoma multiforme stem-like cells accounts for cell clustering and different responses to drugs. Stem Cells Int..

[46] Duman C (2019). Acyl-CoA-binding protein drives glioblastoma tumorigenesis by sustaining fatty acid oxidation. Cell Metab..

[47] Lin H (2017). Fatty acid oxidation is required for the respiration and proliferation of malignant glioma cells. Neuro-Oncology.

[48] Newman JC, Verdin E (2014). beta-hydroxybutyrate: much more than a metabolite. Diabetes Res. Clin. Pract..

[49] Rodrigues LM (2017). The action of beta-hydroxybutyrate on the growth, metabolism and global histone H3 acetylation of spontaneous mouse mammary tumours: evidence of a beta-hydroxybutyrate paradox. Cancer Metab..

[50] Woolf EC, Syed N, Scheck AC (2016). Tumor metabolism, the ketogenic diet and beta-hydroxybutyrate: novel approaches to adjuvant brain tumor therapy. Front. Mol. Neurosci..

[51] Bonuccelli G (2010). Ketones and lactate “fuel” tumor growth and metastasis: Evidence that epithelial cancer cells use oxidative mitochondrial metabolism. Cell Cycle.

[52] Martinez-Outschoorn UE (2012). Ketone body utilization drives tumor growth and metastasis. Cell Cycle.

[53] Whitaker-Menezes D (2011). Evidence for a stromal-epithelial “lactate shuttle” in human tumors: MCT4 is a marker of oxidative stress in cancer-associated fibroblasts. Cell Cycle.

[54] Kang HB (2015). Metabolic rewiring by oncogenic BRAF V600E links ketogenesis pathway to BRAF-MEK1 signaling. Mol. Cell.

