# Supplementary material for: Enhanced fatty acid oxidation provides glioblastoma cells metabolic plasticity to accommodate to its dynamic nutrient microenvironment
Source: Cell Death Dis. 2020 Apr 20;11(4):253. doi: 10.1038/s41419-020-2449-5 (PMC7170895; doi:10.1038/s41419-020-2449-5)
Supplement: Supplementary file 1 — Legends of supplemental figures [file 41419_2020_2449_MOESM1_ESM.docx]

**SUPPLEMENTAL FIGURE LEGENDS**

**Supplementary Figure S1**. Distribution of (A) MGMT promoter methylated (MET) and unmethylated (UNM) and (B) IDH1 mutated (MT) and wild-type (WT) molecular subtypes within acylcarnitine ‘high’ and ‘low’ GBM.

**Supplementary Figure S2**. (A) Schematic representation of FAO dependent and independent OCR measurements. (B) Short-chain acylcarnitines were quantified in MES83 cells +/- etomoxir (40µM; 24 hr; n = 3). (C) Lactate was measured in culture supernatant in MES83 cells treated +/- etomoxir (40µM; 8hr; n = 6). Data represents mean ± SD. ns: not significant.

**Supplementary Figure S3.** (A) Amino acids were quantified in MES83 cells treated with +/- etomoxir (40µM; 24hr; n = 3). (B) Cellular β-HB was measured in MES83 cells plated in regular and nutrient-deprived media followed by treatment with +/- etomoxir (40µM; 8 hr; n = 3-4). Data represents mean ± SD. **p<0.005; ***p<0.0005.

**Supplementary Figure S4. (A)** MES83 cells were pretreated with CPT1A or scrambled siRNA (48 hr; n = 3), followed by treatment in the described conditions. Viable cells were counted using trypan blue after 48 hr. **(B)** CPT1A knockdown was validated using qPCR and data represented as fold change (n = 4-5). Data represents mean ± SD. ***p<0.0005.

**Supplementary Figure S5.** Basal OCR relative to control was measured in MES83 cells treated with indicated compounds for 48 hr (n = 2). Data represents mean ± SD.

**Supplementary Figure S6**. **(A)** MES83 Cells treated with +/- etomoxir (40μM) alone or in combination with β-HB (0.5mM) for 8 hr and indicated acetylated histone proteins were analyzed by western blot. **(B)** Quantification of H3K9 and H3K14 was performed with densitometric analysis, and normalized with total H3 (n = 5). Data represents mean ± SD. P value was calculated on raw data after normalization *p<0.05. Volcano plots were generated using gene expression data from microarray between **(C)** control (n = 3) and etomoxir (40μM; 48hr; n = 3) and **(D)** etomoxir alone or in combination with β-HB (0.5mM; 48hr; n = 4). Non-significant genes are visualized in grey.

**Supplementary Figure S7. (A)** GPR109A (n = 4) and **(B)** GPR43 (n = 5) siRNA knockdown was validated using qPCR. **(C)** MES83 cells pretreated with GPR43 or scrambled siRNA (48 hr; n = 3) were treated with the described compounds for 48 hrs and viable cells were counted using trypan blue after 48 hr (n = 3). Data represents mean ± SD. *p<0.05, **p<0.005; ***p<0.0005.

**Supplementary Figure S8.** p27 expression was evaluated by western blot in MES83 cells **(A/B)** treated with forskolin (40µM; 48 hr, n = 5) and **(C/D)** siRNA knockdown of GPR109A or scrambled siRNA (n = 4). Densitometric quantification of p27 was performed and normalized with tubulin. Data represents mean ± SD. **(E)** MES83 cells were pretreated with p27 or scrambled siRNA (48 hr; n = 3), followed by treatment + / - etomoxir (40 μM; 48hr). Viable cells were counted using trypan blue (n = 3). Data represents mean ± SD. p27 knockdown was validated by western blot (F) and densitometric analysis (n = 3) was performed to evaluate p27 expression (G). densiometric data represents mean ± SD. *p<0.05; **p<0.005; ***p<0.0005

**Supplementary Figure S9.** **(A)** Representative data showing maximal inhibition of ECAR, analyzed using the Seahorse platform at the indicated concentration of 2DG. Data represents mean ± SD of 3-4 wells in a single experiment. **(B)** Apoptosis was analyzed in MES83 cells with Annexin/7AAD (n=4). **(C)** MES83 cells were pretreated with CPT1 or scrambled siRNA (48 hr; n = 3) and then treated +/- with necrostatin-1 (Nec-1; 100µM, 30 min) followed by treatment of 2DG (35mM) alone or in combination with etomoxir (40µM) (n = 3). Non-viable cells were counted after 48 hr. Data represents mean ± SD. ***p<0.0005.

**Supplementary Figure S10.** **(A/B)** MES83 cells were treated with ± etomoxir (40µM), 2DG (35mM) alone or in combination for 48 hr and cell lysates were analyzed for RIP3 expression and expression normalized with tubulin quantified by densitometry (n = 3). **(C/D)** Necroptosis was induced in MES83 cells by treating with the pan-caspase inhibitor Q-VD-OPh (25μM) and BV6 (7.5μM) for 30 min followed by incubation with TNF-α (50ng/ml; 48hr). Molecules involved in necroptosis were visualized using western blot followed by densitometric quantification (n = 3). **(E/F)** Cells were pretreated with RIP1 or scrambled siRNA (24 hr; n = 3) followed by treatment +/- etomoxir (40µM), 2DG (35mM) or the combination. Knockdown was validated by western blot and expression quantified by densitometry, data represents mean ± SD and **(G)** Non-viable cells exposed to these treatment conditions were counted after 48 hr. using trypan blue (n = 3) and represented as mean ± SD. **p<0.005; ***p<0.0005.

**Supplementary Figure S11. (A)** Body weight was measured in mice on the stated days following indicated treatment (n = 10 control and 2-DG; n = 9 etomoxir + 2-DG; n = 6 etomoxir). Data represents mean ± SD of body weight. **(B)** Expression of p27 from intracranial tumors of mice treated with etomoxir and control mice were evaluated using western blot and **(C)** expression was quantified by normalization with tubulin using densitometry and represented as mean ± SD (n = 3) *p<0.005.
