# Supplementary figures and images for: Enhanced fatty acid oxidation provides glioblastoma cells metabolic plasticity to accommodate to its dynamic nutrient microenvironment
Source: Cell Death Dis. 2020 Apr 20;11(4):253. doi: 10.1038/s41419-020-2449-5 (PMC7170895; doi:10.1038/s41419-020-2449-5)

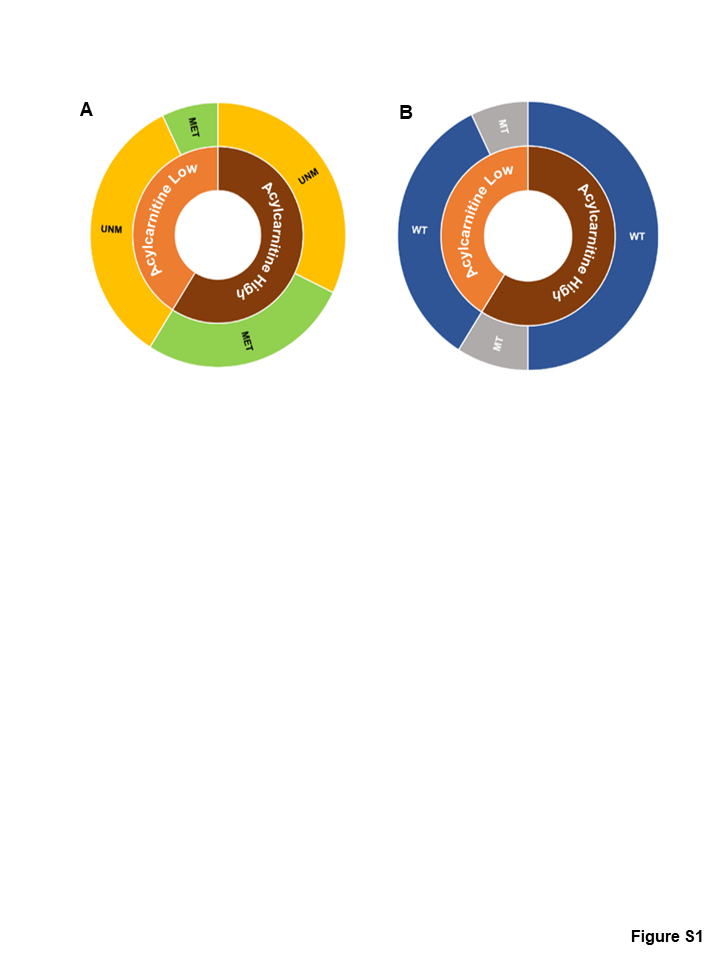

Supplement: Supplementary file 2 — Supplementary Fig. 1 [file 41419_2020_2449_MOESM2_ESM.tif]

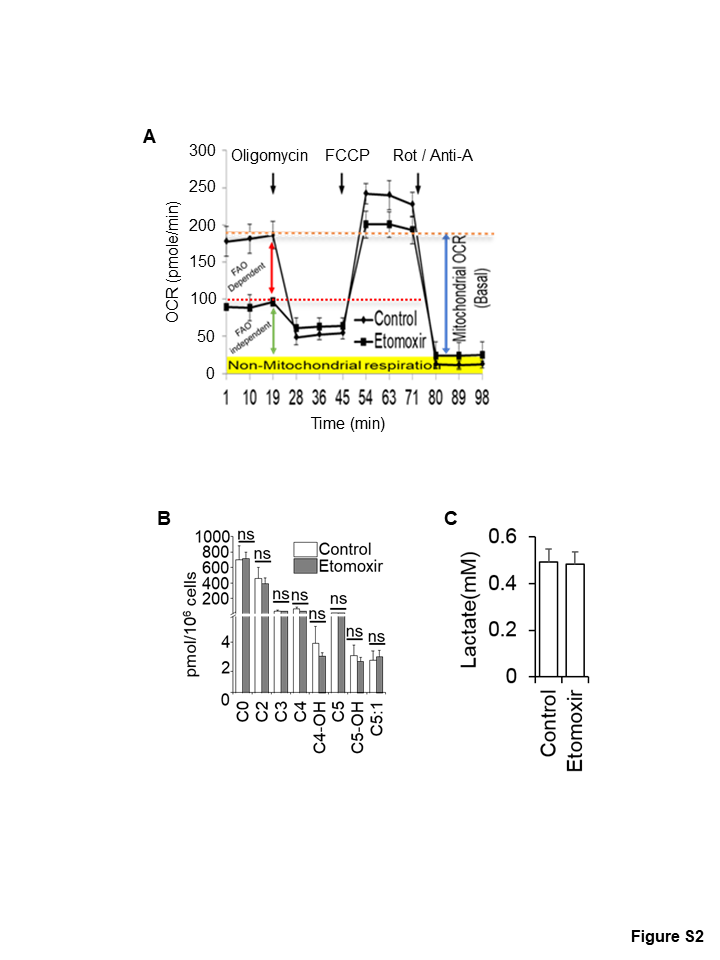

Supplement: Supplementary file 3 — Supplementary Fig. 2 [file 41419_2020_2449_MOESM3_ESM.tif]

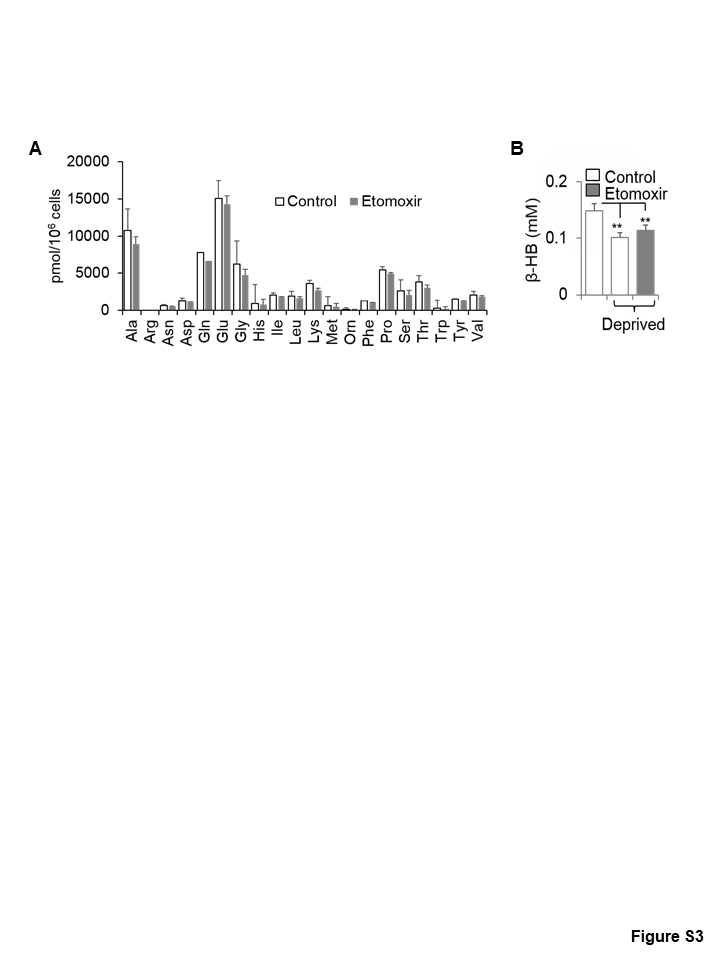

Supplement: Supplementary file 4 — Supplementary Fig. 3 [file 41419_2020_2449_MOESM4_ESM.tif]

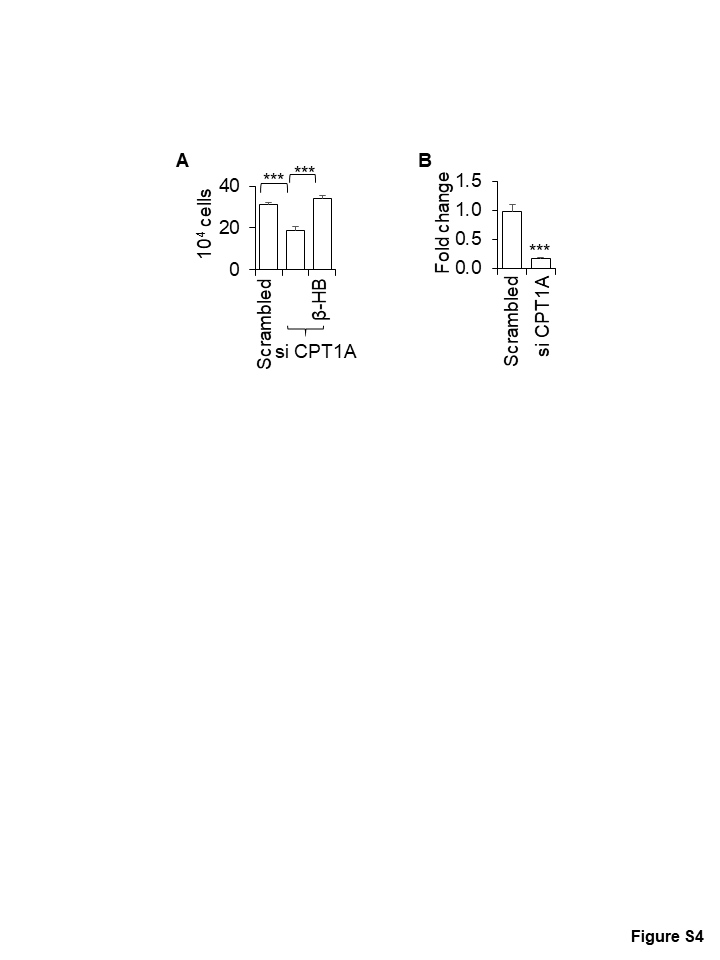

Supplement: Supplementary file 5 — Supplementary Fig. 4 [file 41419_2020_2449_MOESM5_ESM.tif]

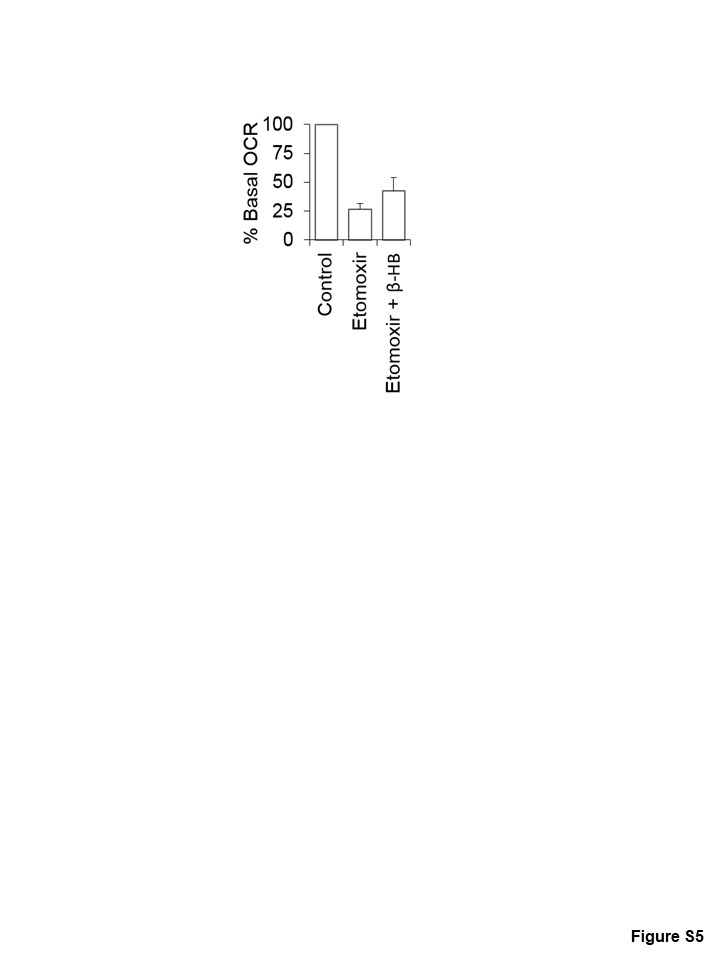

Supplement: Supplementary file 6 — Supplementary Fig. 5 [file 41419_2020_2449_MOESM6_ESM.tif]

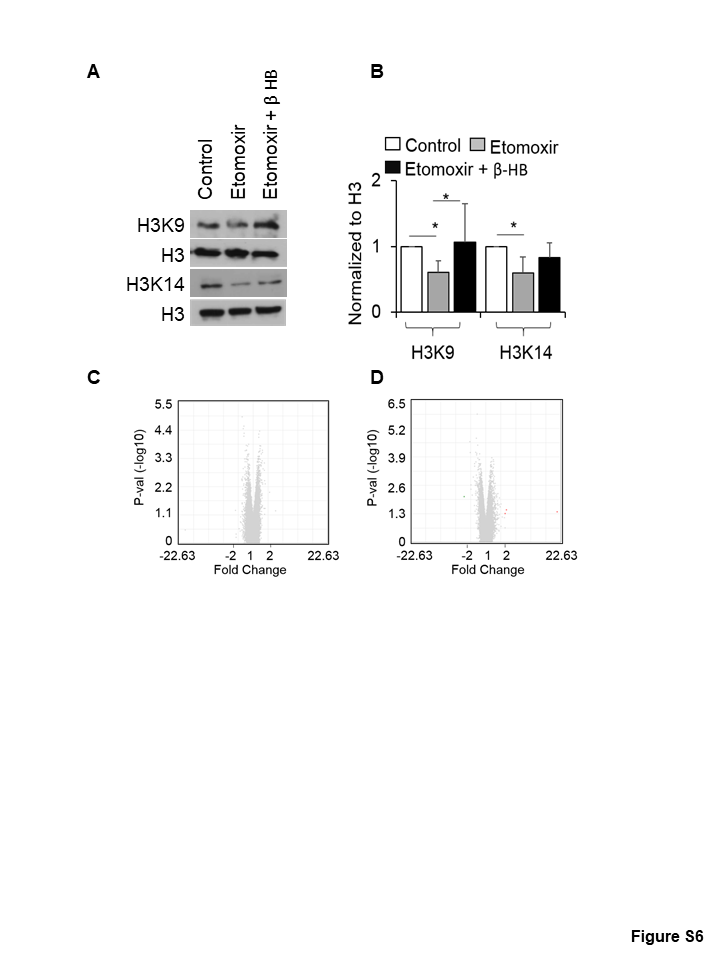

Supplement: Supplementary file 7 — Supplementary Fig. 6 [file 41419_2020_2449_MOESM7_ESM.tif]

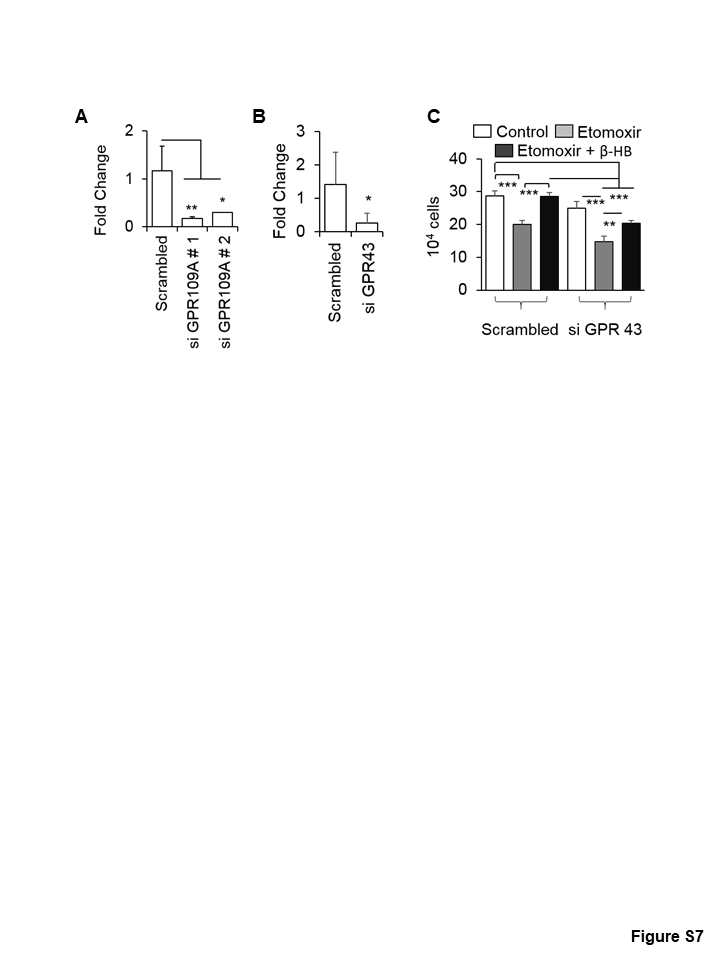

Supplement: Supplementary file 8 — Supplementary Fig. 7 [file 41419_2020_2449_MOESM8_ESM.tif]

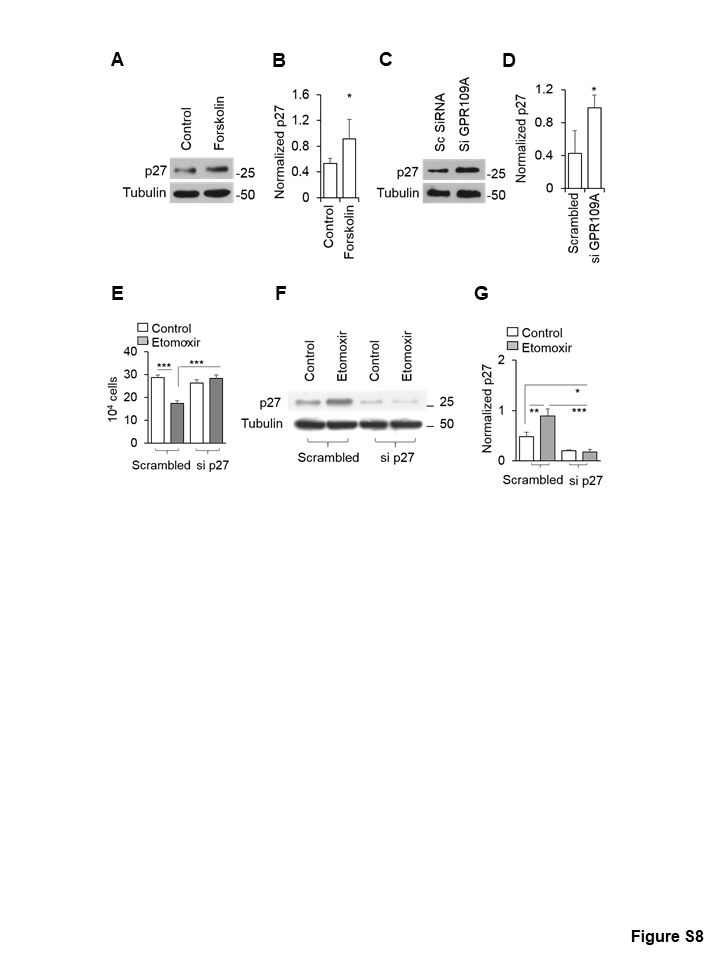

Supplement: Supplementary file 9 — Supplementary Fig. 8 [file 41419_2020_2449_MOESM9_ESM.tif]

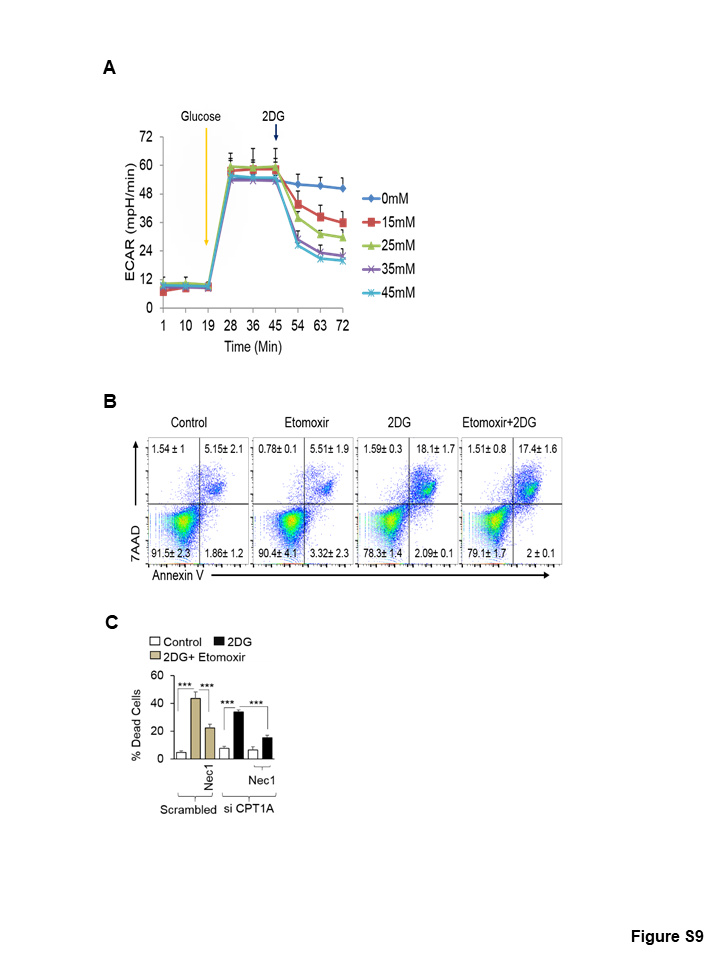

Supplement: Supplementary file 10 — Supplementary Fig. 9 [file 41419_2020_2449_MOESM10_ESM.tif]

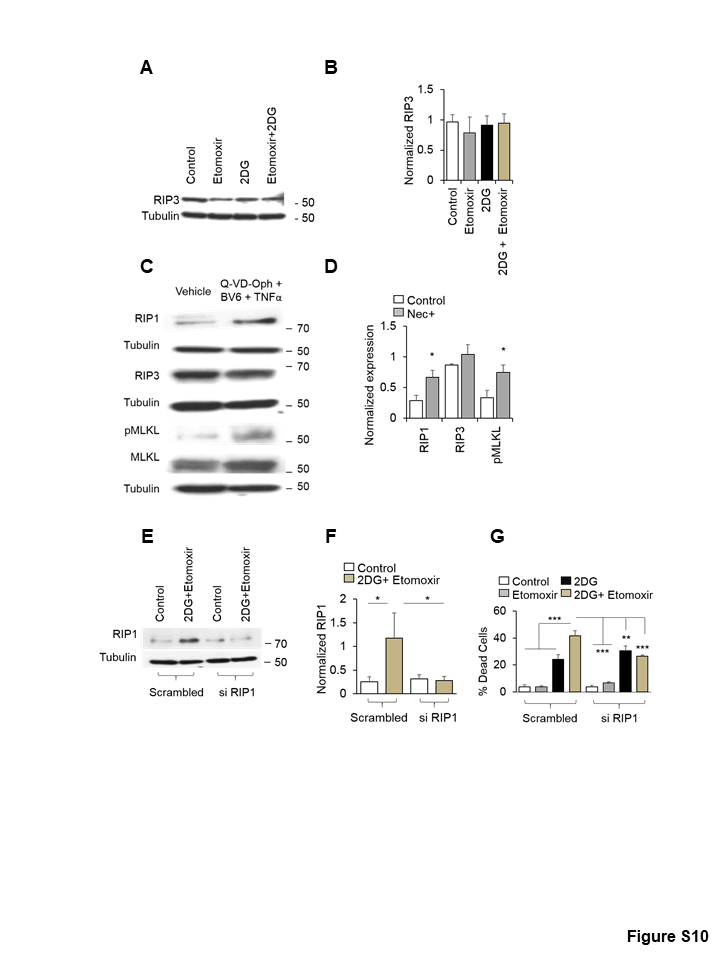

Supplement: Supplementary file 11 — Supplementary Fig. 10 [file 41419_2020_2449_MOESM11_ESM.tif]

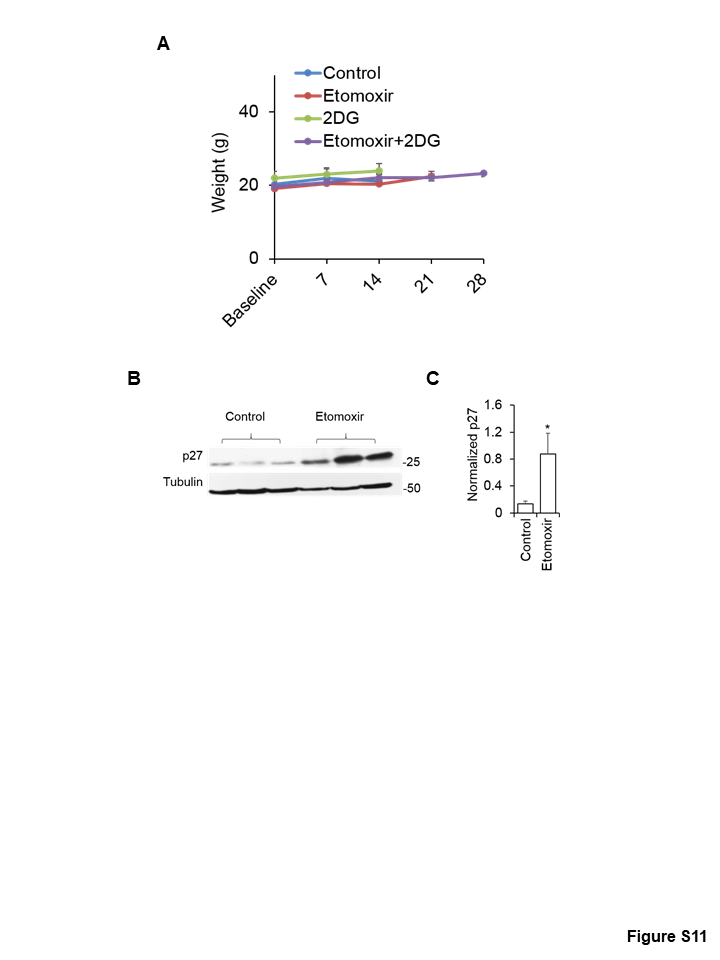

Supplement: Supplementary file 12 — Supplementary Fig. 11 [file 41419_2020_2449_MOESM12_ESM.tif]
